# Structure–Activity Relationship Study of Newly Synthesized Iridium-III Complexes as Potential Series for Treating Thrombotic Diseases

**DOI:** 10.3390/ijms19113641

**Published:** 2018-11-19

**Authors:** Chih-Hao Yang, Chih-Wei Hsia, Thanasekaran Jayakumar, Joen-Rong Sheu, Chih-Hsuan Hsia, Themmila Khamrang, Yen-Jen Chen, Manjunath Manubolu, Yi Chang

**Affiliations:** 1 Department of Pharmacology, Schools of Medicine, College of Medicine, Taipei Medical University, No. 250, Wu Hsing St., Taipei 110, Taiwan; chyang@tmu.edu.tw (C.-H.Y.); sheujr@tmu.edu.tw (J.-R.S.); m120104004@tmu.edu.tw (Y.-J.C.); 2 Graduate Institute of Medical Sciences, College of Medicine, Taipei Medical University, No. 250, Wu Hsing St., Taipei 110, Taiwan; d119106003@tmu.edu.tw (C.-W.H.); jayakumar@tmu.edu.tw (T.J.); d119102013@tmu.edu.tw (C.-H.H.); 3 Department of Chemistry, North Eastern Hill University, Shillong 793022, India; themmilakhamrang@gmail.com; 4 Department of Evolution, Ecology and Organismal Biology, Ohio State University, Columbus, OH 43212, USA; manubolu.1@osu.edu; 5 Department of Anesthesiology, Shin Kong Wu Ho-Su Memorial Hospital, No. 95, Wen Chang Rd., Taipei 111, Taiwan; 6 School of Medicine, Fu-Jen Catholic University, No. 510, Zhong Zheng Rd, Xin Zhuang Dist., New Taipei City 242, Taiwan

**Keywords:** iridium complexes, platelets, ATP, [Ca^2+^]i, signaling cascades, SAR

## Abstract

Platelets play a major role in hemostatic events and are associated with various pathological events, such as arterial thrombosis and atherosclerosis. Iridium (Ir) compounds are potential alternatives to platinum compounds, since they exert promising anticancer effects without cellular toxicity. Our recent studies found that Ir compounds show potent antiplatelet properties. In this study, we evaluated the in vitro antiplatelet, in vivo antithrombotic and structure–activity relationship (SAR) of newly synthesized Ir complexes, Ir-1, Ir-2 and Ir-4, in agonists-induced human platelets. Among the tested compounds, Ir-1 was active in inhibiting platelet aggregation induced by collagen; however, Ir-2 and Ir-4 had no effects even at their maximum concentrations of 50 μM against collagen and 500 μM against U46619-induced aggregation. Similarly, Ir-1 was potently inhibiting of adenosine triphosphate (ATP) release, calcium mobilization ([Ca^2+^]i) and P-selectin expression induced by collagen-induced without cytotoxicity. Likewise, Ir-1 expressively suppressed collagen-induced Akt, PKC, p38MAPKs and JNK phosphorylation. Interestingly, Ir-2 and Ir-4 had no effect on platelet function analyzer (PFA-100) collagen-adenosine diphosphate (C-ADP) and collagen-epinephrine (C-EPI) induced closure times in mice, but Ir-1 caused a significant increase when using C-ADP stimulation. Other in vivo studies revealed that Ir-1 significantly prolonged the platelet plug formation, increased tail bleeding times and reduced the mortality of adenosine diphosphate (ADP)-induced acute pulmonary thromboembolism in mice. Ir-1 has no substitution on its phenyl group, a water molecule (like cisplatin) can replace its chloride ion and, hence, the rate of hydrolysis might be tuned by the substituent on the ligand system. These features might have played a role for the observed effects of Ir-1. These results indicate that Ir-1 may be a lead compound to design new antiplatelet drugs for the treatment of thromboembolic diseases.

## 1. Introduction

Platelets form a plug after their interaction with endothelial matrix proteins to stop excessive bleeding during vascular injury. However, platelet aggregation contributes to thrombotic events, initiating acute coronary syndrome, heart attacks, and strokes [1,2]. After vessel injury, the exposed subendothelial surface makes platelet adhere. Platelet activation and secretion of soluble mediators, such as adenosine 5′-diphosphate (ADP), thromboxane A2 (TXA2), and thrombin, are all involved in the recruitment of other circulating platelets. Currently, numerous antiplatelet drugs are clinically accepted for the treatment and prevention of thrombotic complications. These drugs include acetylsalicylic acid, clopidogrel, eptifibatide, triflusal and tirofiban; however, some have several indemnity effects and battle in long term therapy, such as the known clinical aspirin resistance [3]. Several studies also have reported inter-individual variability in platelet reaction to aspirin and clopidogrel, the well-known oral antiplatelet drugs [4]. The possibility of using antiplatelet agents to substitute oral anticoagulant treatment has been reported in the literature for secondary prevention of further vascular events after limited ischemic stroke due to their lower risk [5].

Platelets also play a serious role in cancer metastasis, including tumor cell migration and invasion [6]. When platelets are activated, they release into the peritumoral space and enhance tumor cell extravasation and metastases [7]. Chronic administration of antiplatelet agents during active malignancy clearly shows the principal role platelets play in maintaining hemostasis. A platelet aggregation inhibitor, cilostazol, reduced pulmonary metastases in a murine model of breast cancer [8]. The authors also observed that liposomal cilostazol decreased ex vivo platelet aggregation and reduced platelet–tumor complex formation in vivo. The existing antiplatelet agents permanently inhibit their target by inhibiting platelet aggregation, however the bleeding risk is still difficult to alleviate. Therefore, efforts are being taken globally to develop new antiplatelet agents with low side effects [9,10].

Organometallic iridium complexes (Ir) have attracted abundant consideration recently due to their unique properties of having rich synthetic chemistry, having variable oxidation states that are prevailing under physiological conditions and being kinetically constant [11]. Furthermore, several organometallic iridium compounds were reported to bind DNA through intercalation [12]. Remarkably, the ligands of most organometallic iridium anticancer complexes are metallocenes, half-sandwich, carbene, CO, or π-ligands [13,14]. We have recently developed some novel Ir-III complexes (Ir-3, Ir-6 and Ir-11) and found they have strong antiplatelet effects with different molecular mechanisms [15,16,17]. From these studies, Shyu et al. found that Ir-3 inhibits platelet activation through the inhibition of signaling pathways, such as the PLCγ2-PKC cascade, and the subsequent suppression of Akt and JNK1 activation, ultimately inhibiting platelet aggregation. In another study, this author found that this compound evidently prolonged the bleeding time in experimental mice, and that this compound plays a crucial role by inhibiting platelet activation via the inhibiting PLCγ2–PKC cascade and the subsequent suppression of Akt and MAPK activation. Recently, our study also demonstrated that Ir-11 increased the bleeding time and reduced mortality related with acute pulmonary thromboembolism [15]. These results encouraged us to work further on Ir complexes on antiplatelet effects. Thus, here we report the synthesis, in vitro antiplatelet, in vivo antithrombotic, and cytotoxicity properties of complexes [Ir(Cp*)(1-(2-pyridyl)-3-phenylimidazo[1,5-*α*]pyridine)Cl]BF_4_ (Ir-1), [Ir(Cp*)(1-(2-pyridyl)-3-(3-nitrophenyl)imidazo [1,5-*α*]pyridine) Cl]BF_4_ (Ir-2) and [Ir(Cp*)(9-[4-(1-pyridin-2-yl-imidazo[1,5-*α*]pyridin-3-yl)-phenyl]-9H-carbazole) Cl]BF_4_ (Ir-4). The structure–antiplatelet activity (SAR) of these compounds have also been analyzed in this paper.

## 2. Results

### 2.1. Effects of Synthetic Ir-III Compounds on Platelet Aggregation In Vitro

**Figure 1:**
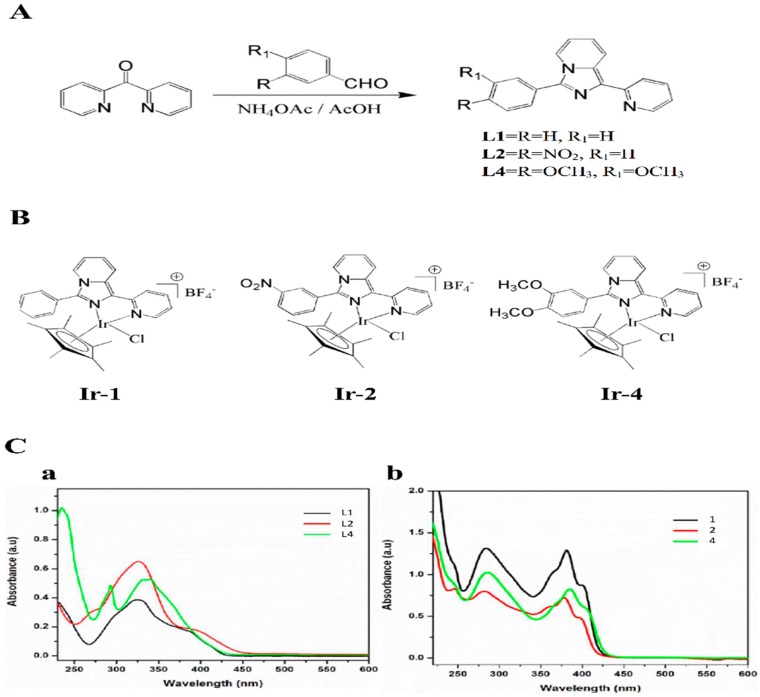
Synthesis of ligands and Ir-III complexes. (**A**) Scheme of synthesis of ligands 1-(2-pyridyl)-3-phenylimidazo[1,5-*α*]pyridine (L1), 1-(2-pyridyl)-3-(3-nitrophenyl)imidazo[1,5-*α*]pyridine (L2) and 9-[4-(1-pyridin-2-yl-imidazo[1,5-*α*]pyridin-3-yl)-phenyl]-9H-carbazole (L4); (**B**) Scheme of synthesis of complexes [Ir(Cp*)(L1)Cl]BF_4_ (Ir-1), [Ir(Cp*)(L2)Cl]BF_4_ (Ir-2) and [Ir(Cp*)(L4)Cl]BF_4_ (Ir-4); (**C**) Absorption spectra of ligands (**a**) and Ir-III complexes (**b**) in acetonitrile.

**Figure 2:**
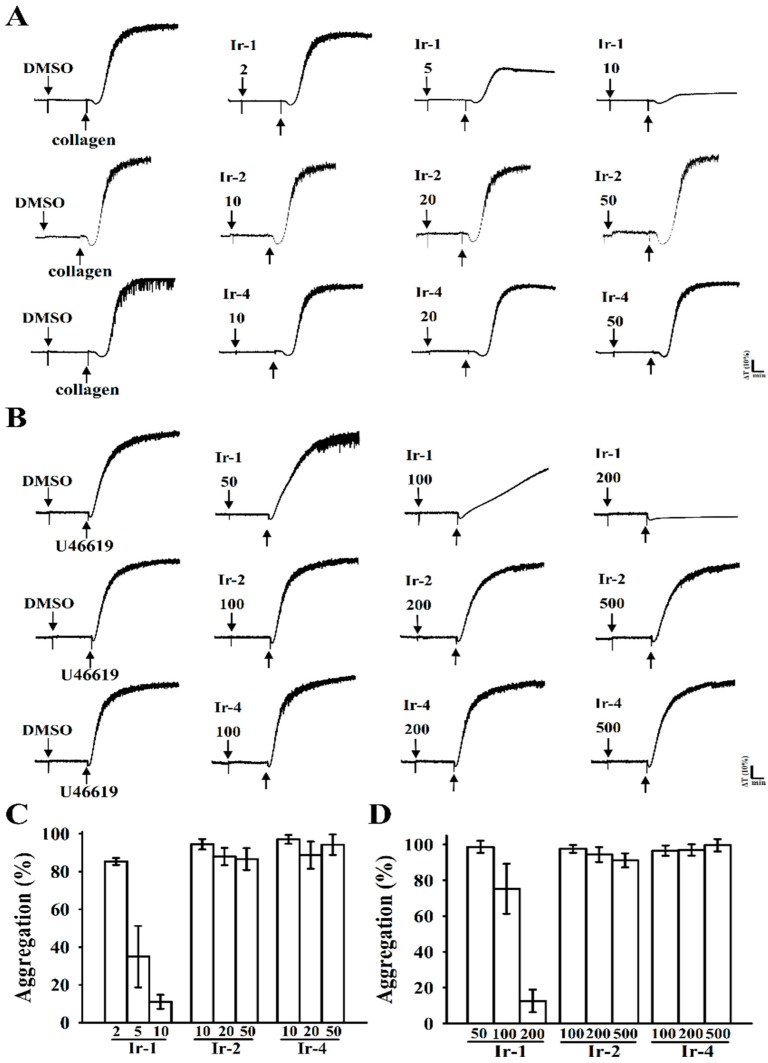
Anti-aggregation activity of iridium complexes (Ir1, Ir-2 and Ir-4) on collagen and U46619-induced platelet aggregation in washed human platelets. (**A**) Washed human platelets (3.6 × 10^8^ cells/mL) were preincubated with the solvent control (0.1% DMSO) or Ir-1 (2–10 μM), Ir-2 and Ir-4 (10–50 μM) for 3 min and then treated with 1 μg/mL collagen for 6 min; (**B**) Washed human platelets were preincubated with the solvent control or Ir-1 (50–200 μM), Ir-2 and Ir-4 (100–500 μM) for 3 min and then treated with 1 μM U46619 for 6 min to stimulate platelet aggregation. Histograms of platelet aggregation in Ir-1, Ir-2 and Ir-4 treated platelets stimulated by collagen (**C**) andU46619 (**D**). Data are presented as means ± standard errors of the means (*n* = 4).

The synthetic Ir-III compounds (Figure 1) were tested for their inhibitory effects on platelet aggregation. Collagen (1 μg/mL) and U46619 (1 µM) stimulated about 85–98% aggregation in washed human platelets. Figure 2A–C shows the in vitro inhibitory effects (%) of various concentrations of Ir-1 (2, 5 and 10 µM), Ir-2 and Ir-4 (10, 20 and 50 µM) against collagen and Ir-1(50, 100 and 200), Ir-2 and Ir-4 (100, 200 and 500 µM) against U46619 induced aggregation in washed human platelets. Among the three compounds tested, Ir-1 had the most potent activity, inhibiting platelet aggregation induced by collagen (11.1 ± 3.7%) and U46619 (12.6 ± 6.3%) at a respective concentration of 10 and 200 µM (Figure 2C,D). Ir-2 and Ir-4 had effects on neither collagen nor U46619 induced platelets. Moreover, in plasma rich platelets (PRP), Ir-1 has almost 20% lower inhibitory effect against collagen-induced aggregation (32.5 ± 2.6%) than in the washed human platelets (11.1 ± 3.7%) data not shown. This result indicate that Ir-1 may slightly bind with plasma proteins and hence it exerts less effect in PRP.

### 2.2. Ir-III Compounds on ATP Release and [Ca^2+^]i Mobilization in Human Platelets

**Figure 3:**
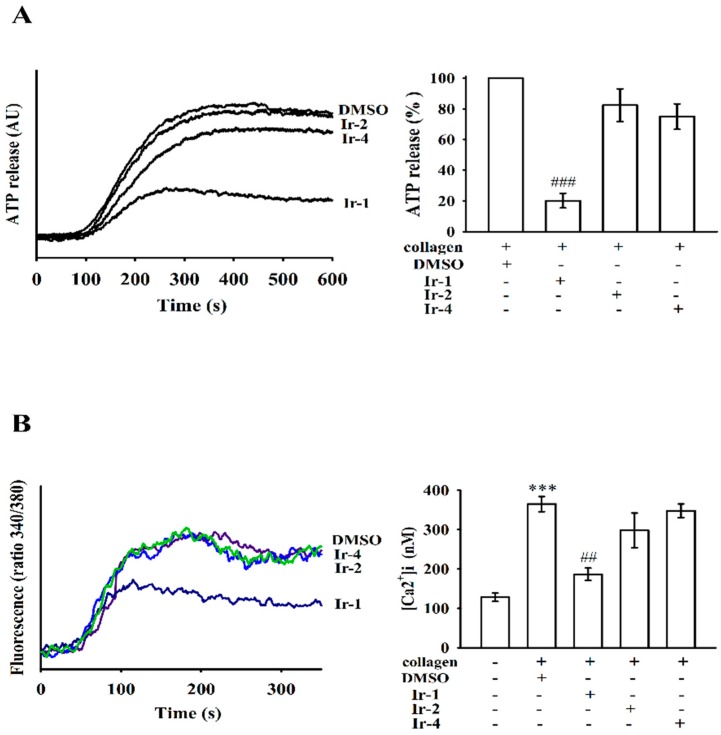
Effects of Ir-1, Ir-2 and Ir-4 on collagen induced ATP release and relative [Ca^2+^]i mobilization in human platelets. Washed human platelets (3.6 × 10^8^ cells/mL) were preincubated with 10 μM Ir-1, Ir-2 and Ir-4 or a solvent control (0.1% DMSO) and subsequently treated with 1 μg/mL of collagen to stimulate ATP release reaction (**A**), and to induce the cytoplasmic influx of Ca^2+^ from intracellular stores (**B**) as described in the Section 4.3 and Section 4.4. Data are presented as the means ± S.E.M. (*n* = 4). *** *p* < 0.001 compared with the DMSO group. ### *p*< 0.001 compared with the collagen induced group. ## *p*< 0.01 compared with the DMSO group.

The release of dense granule content in platelets was assessed through ATP release analysis. Among the three tested compounds, only Ir-1 significantly prevented ATP release from activated platelets stimulated by collagen (Figure 3A). Activation of glycoprotein VI (GPVI), a collagen receptor, leads to quick intracellular calcium mobilization, which is crucial for provoking platelet secretion and aggregation [18]. To investigate the intracellular mobilization of free calcium stores, calcium levels were measured flurometrically using the calcium-sensitive dye, Fura-2 AM. Stimulation of platelets with collagen caused a marked increase of intracellular calcium concentration (Figure 3B). However, Ir-1 inhibited collagen-induced [Ca^2+^]i mobilization on platelets in a manner similar to that observed with the inhibition of ATP release.

### 2.3. Ir-1 Attenuated Collagen Induced P-Selectin Expression without Causing Cytotoxicity

**Figure 4:**
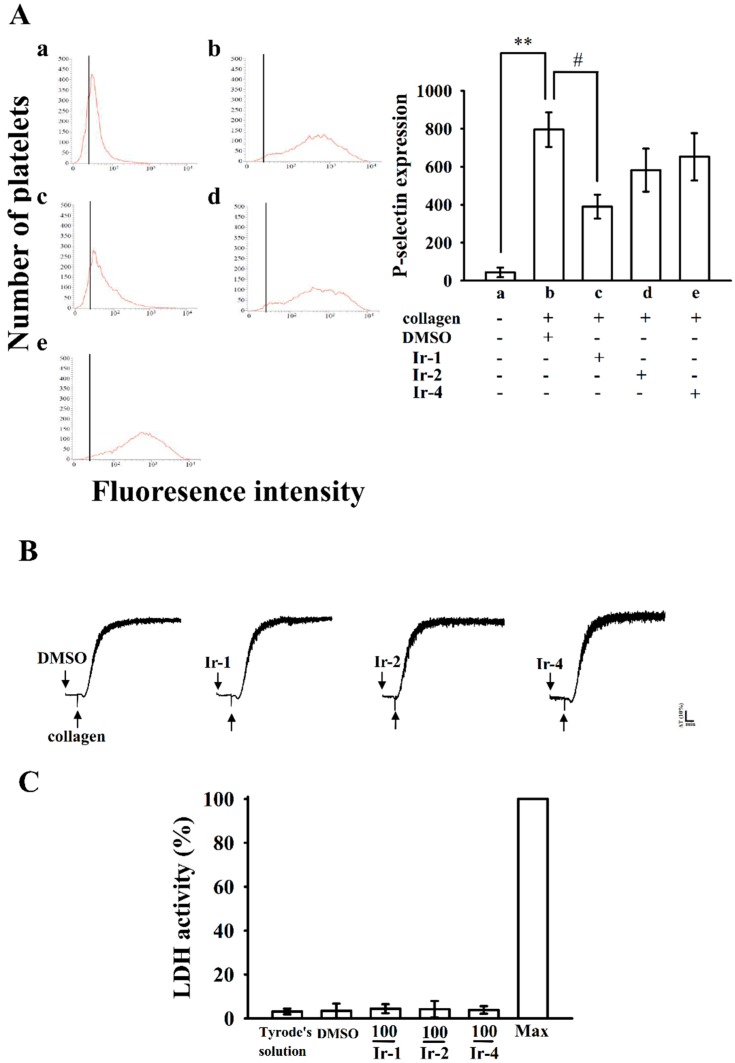
Effects of Ir-1, Ir-2 and Ir-4 on collagen-induced surface FITC-P-selectin expression and cytotoxicity in human platelets. (**A**) Washed human platelets (3.6 × 10^8^ cells/mL) were preincubated with: (**a**) FITC only as a resting control; (**b**) the solvent control (0.1% DMSO); (**c**) Ir-1 (10 μM); (**d**) Ir-2 (10 μM); or (**e**) Ir-4 (10 μM) for 3 min and subsequently treated with 1 μg/mL of collagen to test the direct binding of FITC-P-selectin; (**B**) Washed platelets were preincubated with 0.1% DMSO or Ir-1, Ir-2 and Ir-4 (100 μM) for 10 min and subsequently washed two times with Tyrode’s solution and collagen (1 μg/mL) was then added to trigger platelet aggregation; (**C**) Washed platelets (3.6 × 10^8^/mL) were preincubated with 0.1% DMSO or with 100 µM of Ir-1, Ir-2 and Ir-4 for 20 min, and a 10-µL aliquot of the supernatant was deposited on a Fuji Dri-Chem slide LDH-PIII. Data are presented as the means ± S.E.M. (*n* = 4). ** *p*< 0.01 compared with the DMSO group. # *p*< 0.05 compared with the collagen induced group.

Platelet activation is associated with surface P-selectin expression from α-granules, thus triggering platelet aggregation. In normal platelets, surface P-selectin is positioned on the inner wall of α-granules. Platelet activation results in “membrane flipping”, where the platelet releases α-granules, showing the inner walls of the granules on the outside of the cell [19]. In this study, Ir-2 and Ir-4 only slightly reduced P-selectin expression; however, Ir-1 had a significant effect on P-selectin expression stimulated by 1 μg/mL collagen (Figure 4A).

The aggregation curves of platelets preincubated with 100 µM of Ir-1, Ir-2 and Ir-4 for 10 min and then washed twice with Tyrode’s solution showed no major differences from those of platelets preincubated with the solvent control (0.1% DMSO) (Figure 4B), demonstrating that the effects of Ir complexes on platelet aggregation are alterable and noncytotoxic. Additionally, the LDH study showed that the tested Ir complexes (100 µM) incubated with platelets for 20 min did not significantly increase LDH activity or exert cytotoxic effects on platelets (Figure 4C), demonstrating that Ir-compounds do not disturb platelet permeability or induce platelet cytolysis.

### 2.4. Ir-1 Tempered MAPK and Akt/PKC Phosphorylation

**Figure 5:**
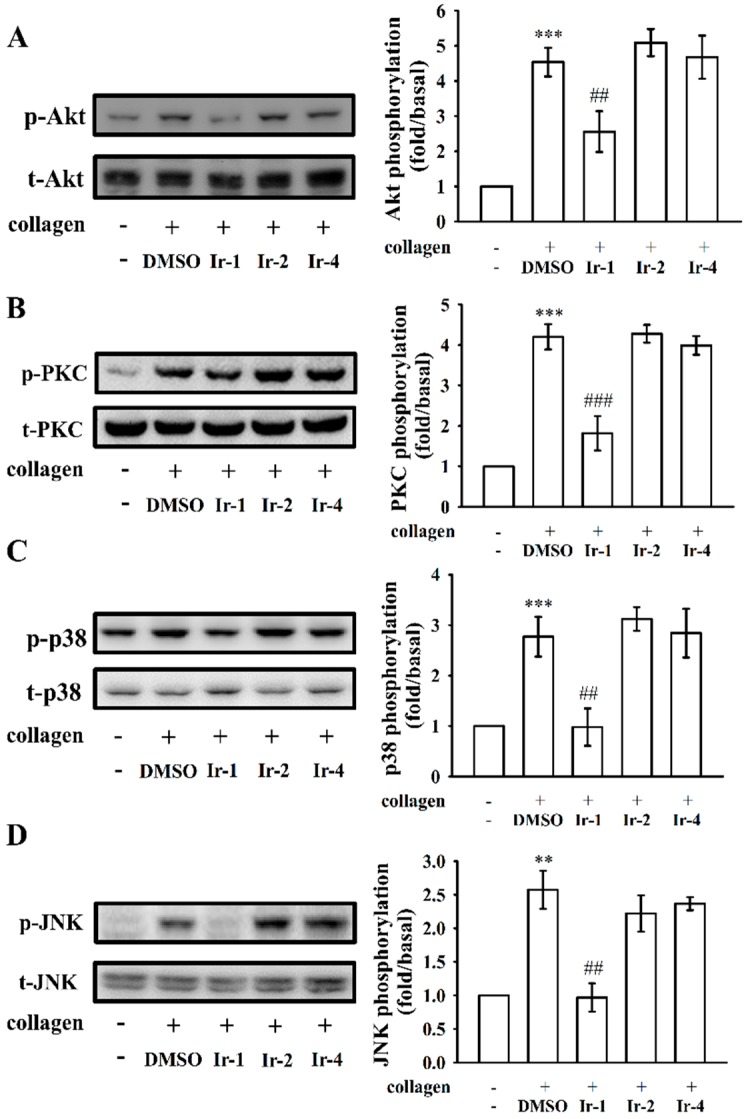
Effects of iridium-III complexes on the phosphorylation of Akt/PKC and MAPKs induced by collagen in human platelets. Washed platelets (1.2 × 10^9^ cells/mL) were incubated with solvent control (0.1% DMSO) or 10 μM Ir-1, Ir-2 and Ir-4 and then treated with 1 μg/mL collagen to induce platelet activation. The subcellular extracts were analyzed by Western blotting for the phosphorylation of: Akt (**A**); PKC (**B**); p38MAPK (**C**); and JNK (**D**). Data are presented as the mean ± SEM (*n* = 4). ** *p* < 0.01 and *** *p* < 0.001 compared with the normal group, ## *p* < 0.01 and ### *p* < 0.001 compared with the collagen induced group.

MAPKs, such as P38MAPK, JNK and ERK, are expressed in platelets and different agonists can activate them. Phosphorylation of these signaling molecules plays a central role in activating platelet granule secretion [20]. Consequently, to clarify the mechanism underlying the effects of tested Ir-complexes on agonist-induced platelets, we studied the Akt/PKC and MAPK pathway (Figure 5A–D). Among the tested compounds, only Ir-1 significantly attenuated the phosphorylation of Akt/PKC and MAPK molecules p38 and JNK (Figure 5). Ir-2 and Ir-4 had no effect on these molecules.

### 2.5. Ir-1 Inhibits Platelet Aggregation via Interrupting the Association of JAQ-1 with GPVI

**Figure 6:**
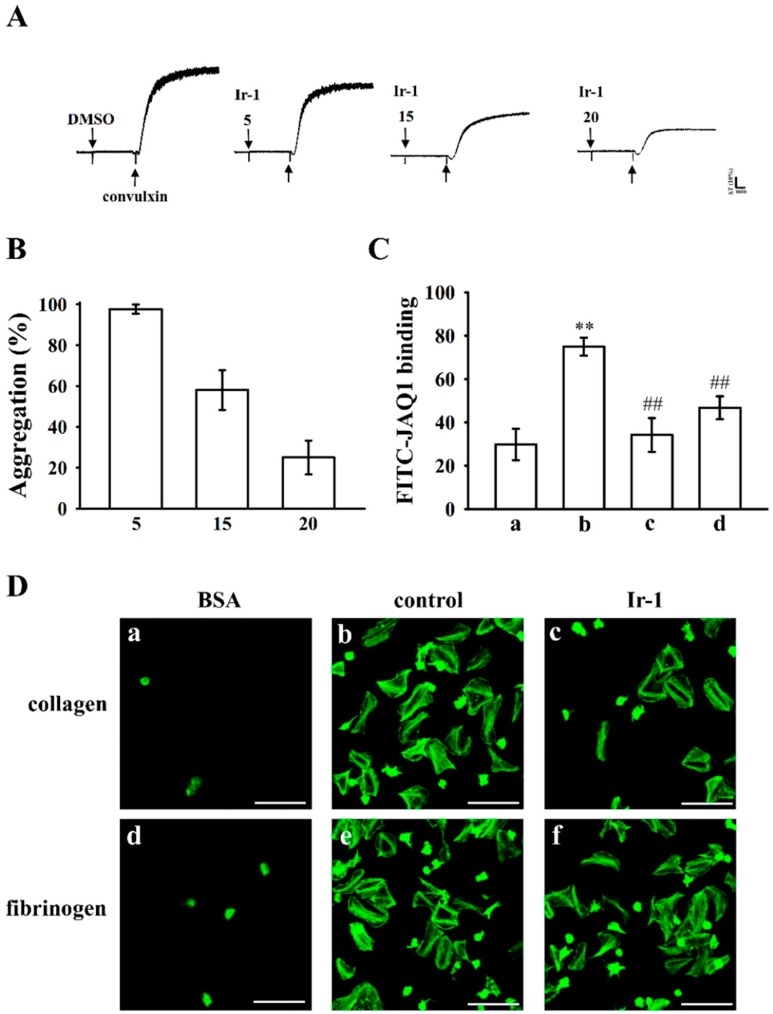
Effects of Ir-1 on convulxin-induced platelet aggregation, adhesion and spreading in human platelets. (**A**) Washed platelets (3.6 × 10^8^ cells/mL) were preincubated with Ir-1 (5–20 μM) or the solvent control (0.1% DMSO), followed by the addition of 10 ng/ml of convulxin; (**B**) Concentration-response (%) histograms of Ir-1 in inhibition of convulxin-induced platelet aggregation; (**C**) Statistical graphs represent the platelets in the presence of: (a) FITC only (background); (b) FITC-JAQ1 mAb (1 μg/mL); (c) preincubated with convulxin (10 ng/mL); and (d) Ir-1 (20 μM), followed by the addition of FITC-JAQ1 mAb (1 μg/mL); (**D**) Washed human platelets were allowed to spread on: (**a**,**d**) bovine serum albumin (BSA); (**b**,**c**) collagen; and (**e**,**f**) fibrinogen-coated surfaces at 37 °C for 45 min in the presence of (**b**,**e**) the solvent control (0.1% DMSO) or (**c**,**f**) Ir-1 (10 μM, 5 min, at 37 °C) and then fixed with paraformaldehyde to stop spreading. Platelets were subsequently labeled with FITC-conjugated phalloidin and photographed under a confocal microscope (scale bar = 10 μm). Data are presented as means ± standard errors of the means (*n* = 3). ** *p* < 0.01, ## *p* < 0.01 compared with the control group.

Integrin α2β1 and GPVI are major collagen receptors that mediate platelet adhesion and aggregation. To investigate whether Ir-1 inhibits platelet aggregation via GPVI, we used convulxin, a GPVI agonist, which is purified from the venom of *Crotalusdurissus terrificus* to induce platelet aggregation. The results show that treatment with Ir-1 (5–20 μM) significantly inhibited platelet aggregation stimulated with 10 ng/mL of convulxin (Figure 6A,B).

The above result was further confirmed by flow cytometry analysis that Ir-1 inhibits platelet activation via interrupting the convulxin and GPVI interaction on the platelet membrane. The results revealed that the relative fluorescence intensity of FITC-JAQ1 (mAb raised against GPVI; 1 μg/mL) bound to the platelets was significantly higher than that of the resting counterparts (Figure 6C). This FITC-JAQ1 mAb binding significantly diminished in the presence of convulxin (10 ng/mL) or Ir-1 (20 μM). Overall, although these results may suggest that Ir-1 could interrupt the association between JAQ-1 and GPVI to its antiplatelet mechanism, however the possibility of other unidentified mechanisms involved in this reaction needs to be clarified in future studies.

### 2.6. Ir-1 Restricts Cell Adhesion and Spreading on Immobilized Collagen

As shown in Figure 6Da,b, platelets staining with FITC-conjugated phalloidin demonstrated that platelets adhered to immobilized collagen were significantly more than immobilized BSA. In addition, Ir-1-treated platelets had lower adhesion and spreading on immobilized collagen than did 0.1% DMSO-treated platelets (Figure 6Dc). Control platelets (0.1% DMSO, data not shown, *n* = 3) were fixed to immobilized collagen normally, whereas Ir-1-treated platelets showed less adhesion to the collagen-coated surface. Ir-1 inhibited platelet adhesion by approximately 49.7 ± 1.1%. The percentage of spreading platelets treated with Ir-1 was approximately 34.3 ± 3.2%. The platelet adhesion and spreading between 0.1% DMSO-treated platelets and Ir-1 on immobilized fibrinogen were no significant.

### 2.7. Ir-1 on Closure Time

**Figure 7:**
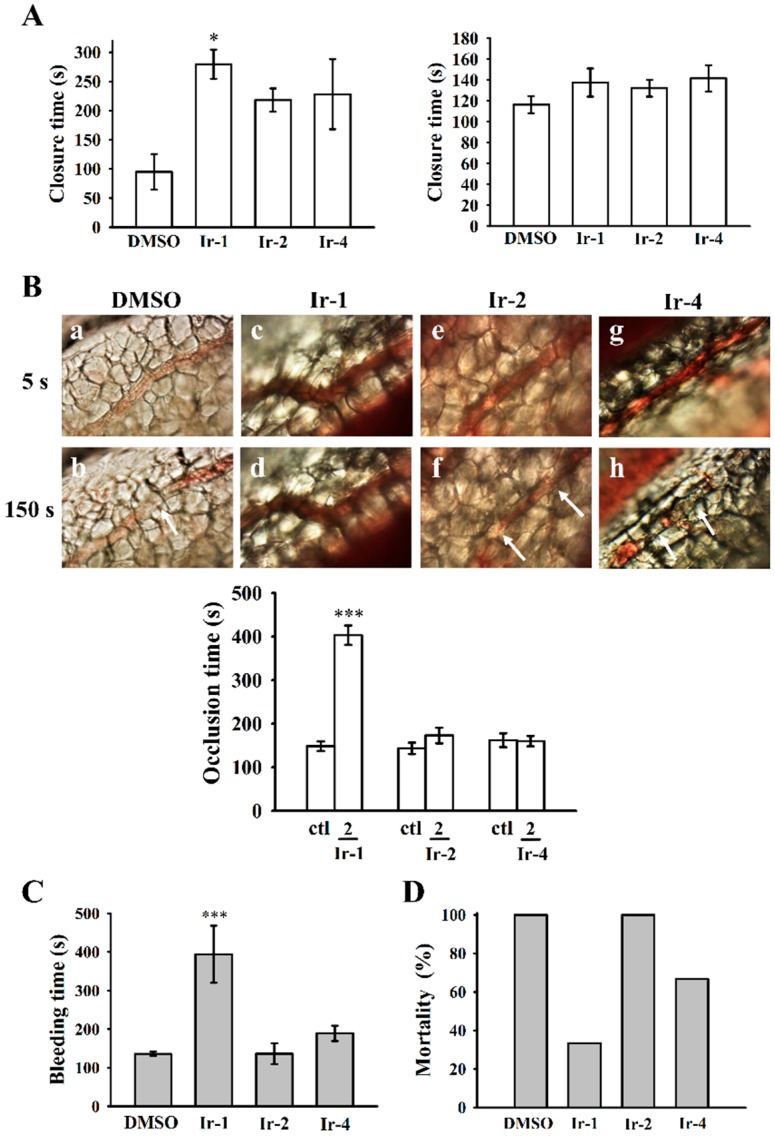
Effects of iridium complex on PFA-100 closure time and thrombotic platelet plug formation in the mesenteric venules, bleeding time and ADP induced pulmonary thrombosis in mice. (**A**) Shear-induced platelet plug formation in human whole blood was determined by recording the closure time of C-ADP and C-EPI-coated membranes, as described in the Section 4.8; (**B**) Mice were administered an intravenous bolus of the solvent control (ctl; 0.1% DMSO) or Ir-1, Ir-2 and Ir-4 (2 mg/kg), and the mesenteric venules were irradiated to induce microthrombus formation (occlusion time). Microscopic images (400× magnification) of 0.1% DMSO-treated controls (a,b) and the 2 mg/kg Ir-1 (c,d), Ir-2 (e,f) and Ir-4 (g,h) treated groups were recorded at 5 s (a,c,e,g) and 150 s (b,d,f,h) after irradiation. The photographs are representative of six similar experiments. The arrow indicates platelet plug formation; (**C**) The bleeding time was measured through transection of the tail in mice after 30 min of administering 2 mg/kg Ir-1, Ir-2 and Ir-4 intraperitoneally; (**D**) Effects of Ir-1, Ir-2 and Ir-4 on ADP-induced pulmonary thrombosis in mice. ADP (700 mg/kg) was injected in the tail vein to induce acute pulmonary thrombosis. The survival rate was determined after ADP administration. The survival rate was evaluated using the Kaplan–Meier survival method (*n* = 8). Data are presented as the mean ± SEM (*n* = 8). * *p* < 0.05 and *** *p* < 0.001, compared with the 0.1% DMSO-treated group.

Platelet aggregation in response to ADP and epinephrine (EPI) was recorded using a platelet function analyzer (PFA-100) with both collagen-ADP (C-ADP) and collagen epinephrine (C-EPI) cartridges. The closure time (CT) of the C-ADP-coated membrane in whole blood treated with 10 µM Ir-1 was significantly increased (279.6 ± 25.0 s) when compared to the solvent control DMSO (95.0 ± 30.6 s), however, the CT was not prolonged in C-EPI-coated membrane (Figure 7A). Ir-2 and Ir-4 did not prolong either C-ADP or C-EPI closure times as the levels remained within normal ranges as established by the PFA-100 test.

### 2.8. Effects of Ir Complexes on Occlusion Time and Bleeding Time

Platelet-rich thrombus formation in mesenteric venules was observed in fluorescein sodium and filtered light irradiated ICR mice. The time lapse for inducing occlusion in the irradiated vessel was 148.3 ± 11.2 s (*n* = 8). From the data shown in Figure 7B, among the tested Ir-III complexes, Ir-1 (2 mg/kg) expressively prolonged the occlusion time to 403.1 ± 22.3 s (*n* = 8). The thrombotic platelet plug formation was detected in mesenteric microvessels at 150 s, whereas it was noted at 5 s after irradiation in the solvent-treated group. Platelet plug formation was not observed at either 5 or 150 s after irradiation in mice that had been treated with Ir-1 (2 mg/kg). The rate of blood flow in the solvent-treated venule was slower than that of the Ir-1-treated venule, as the platelet plug converted at 150 s (Figure 7B).

The tail bleeding time of mice that were intraperitoneally injected with DMSO was measured as 135.8 ± 5.5 s (*n* = 8). A dose of 2 mg/kg Ir-1 treated mice showed a significant prolongation (394.0 ± 73.9 s) of the bleeding time 30 min after injection. Ir-2 or Ir-4 did not prolong the bleeding time, as their effects were similar to that in control mice (Figure 7C).

### 2.9. Ir-Complexes on ADP-Induced Acute Pulmonary Thromboembolism

An in vivo study was performed to test the effect of Ir-complexes on inhibiting acute pulmonary embolism mortality in mice. As shown in Figure 7D, a dose of 2 mg/kg Ir-1 prominently lowered the mortality rate in mice, from 100% to 33.3% in those that had been challenged with ADP (700 mg/kg) indicating that Ir-1 efficiently stops acute pulmonary thromboembolism in vivo.

## 3. Discussion

In this study, three iridium (III) complexes, [Ir(Cp*)(1-(2-pyridyl)-3-phenylimidazo[1,5-*α*]pyridine)Cl]BF_4_ (Ir-1), [Ir(Cp*)(1-(2-pyridyl)-3-(3-nitrophenyl)imidazo [1,5-*α*]pyridine) Cl]BF_4_ (Ir-2) and [Ir(Cp*)(9-[4-(1-pyridin-2-yl-imidazo[1,5-*α*]pyridin-3-yl)-phenyl]-9H-carbazole) Cl]BF_4_ (Ir-4) with 1-(2-pyridyl)-3-phenylimidazo[1,5-*α*]pyridine, 1-(2-pyridyl)-3-(3-nitrophenyl)imidazo[1,5-*α*]pyridine and 9-[4-(1-pyridin-2-yl-imidazo[1,5-*α*]pyridin-3-yl)-phenyl]-9H-carbazole as ligands, respectively, were synthesized and evaluated for their antiplatelet activities. Among them, Ir-1 exhibited the most potent antiplatelet activity against collagen and U4661 induced human platelet aggregation, while Ir-2 and Ir-4 had no effect. Ir-1 also exhibited potent in vivo antithrombotic effects. Overall, these findings highlight the exciting potential of iridium (III) compounds to be developed as effective antiplatelet drugs for the treatment of thromboembolic diseases. In addition, Ir-1 may be considered as a leading compound to design a new class of antiplatelet drugs.

Ligand-gated Ca^2+^-permeable ion channels are express on the platelet surface and provide a rapid route for Ca^2+^ entry to release ATP from damaged vascular cells or from activated platelets and other blood cells [21,22]. These channels shown to increase in vitro platelet [Ca^2+^]i responses following stimulation by several major hemostatic agonists, including ADP, collagen, thrombin and thromboxane A2, and also aggravate in vivo thrombosis [23]. Evaluation of platelet-surface P-selectin in activated platelets can be used as a substitute to measurement of platelet aggregation for analysis of platelet defects and in monitoring the effectiveness of drug treatments [24,25]. Therefore, inhibition of [Ca^2+^]i mobilization, ATP production and platelet-surface P-selectin is important for appraising the effectiveness of the antiplatelet drugs. Our previous studies found some newly synthesized Ir-III compounds, such as [Ir (Cp*) 1-(2-pyridyl)-3-(3-methoxyphenyl)imidazo[1,5-*α*]pyridine Cl]BF4 (Ir-3), [Ir(Cp*)1-(2-pyridyl)-3-(4-dimethylaminophenyl)imidazo[1,5-*α*]pyridine Cl]BF4 (Ir-6) and [Ir(Cp*)1-(2-pyridyl)-3-(2-hydroxyphenyl)imidazo[1,5-*α*]pyridine Cl]BF4 (Ir-11), potently inhibit collagen-activated [Ca^2+^]i mobilization, ATP production and platelet-surface P-selectin expression in washed human platelets. Consistent with these results, this work observed that, among the three tested Ir-III complexes (Ir-1, Ir-2 and Ir-4), Ir-1 effectively inhibited collagen stimulated [Ca^2+^]i mobilization, ATP production and P-selectin expression. These results indicate that inhibition of these granular substances may contribute to the important antiplatelet effects of Ir-1 compounds.

Due to the integrity of the cell membrane after the release of LDH, a decrease in LDH release from the cytoplasm and a supplement of enough oxygen for platelet metabolism are important factors for improving survival and quality of platelet concentrate during storage [26]. In this study, the LDH enzyme activity was expressively increased, as evidenced by a maximal value (MAX) of LDH in the sonicated platelets, which is used as a positive control as compared with the DMSO, Tyrode’s solution and Ir-III complex treated groups. It seems that Ir-III complexes-treated platelets had no membrane damage and thus they could have beneficial effects on the platelets quality.

Akt pathway had reported in the activated platelets by GPVI via the modulation of the serine/threonine kinase, Akt [27]. This molecule was found to be phosphorylated by PKC and Ca^2+^/calmodulin-dependent protein kinase [28]. Akt deficient mice showed impaired platelet activation induced by collagen [29]. MAPKs are reported to exist in platelets and be intricately involved in the action of numerous anti-platelet agents [30]. Studies with inhibitors have established that MAPKs contribute critically to platelet reactions in different agonists [31]. Several inflammatory cytokines and stress inducers activate JNK1/2 and p38 MAPK that lead to cellular apoptosis [32]. A study had also shown that JNK deficient platelets are directly correlated with increased bleeding time, decreased integrin *α*IIb*β*3 activation, and severe granule secretion [31]. Moreover, p38 MAPK is associated with thrombus formation, as demonstrated in p38^+/−^ mice in a model of ferric chloride-induced carotid artery occlusion [21]; the removal of JNK1 also weakens in vitro collagen induced platelet aggregation and granule release and in vivo thrombus formation [31]. Consequently, the inhibition of Akt, PKC and JNK/p38 phosphorylation can be considered to be playing an important role in the antiplatelet action of drugs or compounds. This hypothesis is consistent with the current study that among the three tested Ir-III complexes, Ir-1 had potently suppressed collagen-induced Akt, PKC, p38 and JNK phosphorylation, while Ir-2 and Ir-4 did not affect these molecules. These findings support that Ir-1 could reduce collagen-induced platelet activation, including granule release, [Ca^2+^]i mobilization, and platelet aggregation, partly through the inhibition of Akt, PKC, p38 and JNK activation.

PFA-100, a new method of measuring the primary hemostasis induced by platelet adhesion and aggregation under shear stress conditions, has recently been familiarized. This test measures the time of platelet plug formation, called closure time [33]. The clinical effectiveness of PFA-100 has been confirmed in various platelet disorders, including the platelet dysfunction especially in uremic patients [34]. In addition, PFA-100 has evidenced to be useful in measuring surgical bleeding risk in patients who had oral surgery and other surgical procedures [33,35]. Further, some reports designate that the use of PFA-100 as perioperative screening test is connected with an increase in the use of desmopressin prophylaxis that still was not supportive in reducing the rate of bleeding complications [36]. In the present study, we analyzed the PFA-100 (C-EPI and C-ADP closure times) for the risk of bleeding complications after Ir-III complex treatment. Our results did show significant differences in the closure time values of the C-ADP cartridge of the PFA-100 test in Ir-1 treated platelets. In addition, we were not able to detect any significant changes in C-EPI cartridge of the PFA-100 closure time values.

Antiplatelet drugs are typically used in thrombogenic diseases, but acute intense thrombocytopenia is a documented complication of treatment with antiplatelet drug (e.g., GPIIb/IIIa inhibitors), and the reason is not yet fully identified. Thrombocytopenia may be related with a variety of conditions, and life-threatening bleeding is one of its worst side effects. Since GPVI expression scarcity is only associated with faintly prolonged bleeding times, it is worth developing novel GPVI antagonists for thrombogenic diseases. In this study, Ir-1 significantly tempered thrombus formation in two in vivo models of fluorescein sodium-induced platelet thrombus formation in the mesenteric microvessels and ADP-induced pulmonary thrombosis in mice. In addition, Ir-1 did not affect normal hemostasis without inducing mortality in mice. These results suggest that Ir-1 may recognize as safe antithrombotic agent. In this study, FITC-JAQ1 mAb bound to the platelets was significantly higher than that of the resting platelets. This FITC-JAQ1 binding significantly reduced by convulxin or Ir-1, indicating that Ir-1 may interrupt the association between the JAQ-1 and the collagen receptor GPVI on human platelets. As shown in Figure 6D, platelet spreading on fibrinogen-coated membrane seems visually to be attenuated after platelet pretreatment by Ir-1 (Figure 6D, compare e to f) although the difference is not significant. This result may clarify by a report that GPVI dimers represent a significant part of GPVI on the surface of resting platelets and are able to bind fibrinogen [37]. In mice naturally deficient in GPVI, the tail bleeding time is usually not prolonged, in contrast with the noticed in vivo effect of Ir-1. This phenomenon may suggest that Ir-1 has additional effects, besides the collagen-GPVI axis.

Generally, addition of polar substituents into the coordinated benzene ring lowers cytotoxicity or biological activity [38]. This hypothesis was confirmed from the IC_50_ values of monosubstituted benzenes: OPh < H (benzene) < CONH2, COOEt, COPh, COOMe, Br, CH2OH. Activities ranged from moderate (18 µM) to inactive (> 100 µM). In this study, based on the previous literature, these type of Ir-III (Ir-1, Ir-2 and Ir-4) compounds could interact with biological systems after hydrolysis, and the chloride ions in the complexes may be replaced by water molecule (as in cisplatin). The rate of hydrolysis may be tuned by the substituent on the ligand system. Mostly, the complexes hold increase in rate of hydrolysis display greater cytotoxicity than the compounds, which are inactive or weakly active with less hydrolyzing properties. A study showed that bovine, ovine, and caprine κ-casein and their hydrolysates exhibit platelet inhibitory activities due to their potent hydrolytic behaviors [39]. A previous important study also established that carbamoylpiperidine and nipecotoylpiperazine derivatives augment desired antithrombotic effects because of their increased levels of hydrophobicity [40]. Therefore, we postulated that the difference in the antiplatelet activities of complexes Ir-1, Ir-2, and Ir-4 is mainly dependent on the respective substitution on the phenyl (no substitution), nitrophenyl (electron withdrawing substitution) and dimethoxy (electron donating substitution) groups on the ring of the iridium ligands. Here, the absence of substitution on phenyl group in Ir-1 could increase the rate of hydrolysis on its ligand system and might play a role in its observed antiplatelet and antithrombotic effects.

## 4. Materials and Methods

### 4.1. Reagents

Thrombin, U46619, heparin, collagen, fibrinogen, FITC-phalloidin and bovine serum albumin (BSA) were purchased from Sigma (St. Louis, MO, USA). Fura-2AM was purchased from Molecular Probes (Eugene, OR, USA). An anti-phospho-p38 mitogen-activated protein kinase (MAPK) Ser^182^ monoclonal antibody (mAb) and convulxin were purchased from Santa Cruz Biotechnology (Santa Cruz, CA, USA). Anti-p38 MAPK, anti-phospho-c-Jun N-terminal kinase (JNK) (Thr^183^/Tyr^185^), anti-phospho-(Ser) protein kinase C (PKC) substrate (pleckstrin; p-p47), and anti-JNK polyclonal antibodies (pAbs) were purchased from Cell Signaling (Beverly, MA, USA). Anti-phospho-protein kinase B (Akt) (Ser^473^) and anti-Akt mAbs were purchased from Biovision (Mountain View, CA, USA). Fluorescein isothiocyanate (FITC)-labeled anti-GPVI (JAQ1) mAb was obtained from Emfret Analytics (Würzburg, Germany). Hybond-P polyvinylidene fluoride (PVDF) membranes, an enhanced chemiluminescence Western blotting detection reagent, and antibodies, namely horseradish peroxidase (HRP)-conjugated donkey anti-rabbit immunoglobulin G (IgG), and sheep anti-mouse IgG, were purchased from Amersham (Buckinghamshire, UK). A fluorescein isothiocyanate (FITC) anti-human CD42P (P-selectin) mAb was obtained from BioLegend (San Diego, CA, USA).

### 4.2. Synthesis of Ligands and Ir-III Complexes

Synthesis and characterization of the ligands 3-phenyl-1-pyridin-2-yl-imidazo[1,5-*α*]pyridine(L1), 3-(3-nitrophenyl)-1-pyridin-2-yl-imidazo[1,5-*α*]pyridine (L2) and 3-(3,4-dimethoxyphenyl)-1-pyridin-2-yl-imidazo[1,5-*α*]pyridine (L4) are similar to that of the reported method by Wang et al. [41], as shown in Figure 1A. According to the method described [42], the iridium complexes were prepared by mixing the ligands with [Ir(Cp*)(Cl)_2_]_2_ (Figure 1B). A representative synthetic scheme for the metal complexation is illustrated below for TIr-1. The absorption spectra of ligands and complexes in acetonitrile is shown in Figure 1Ca,b.

[Ir (Cp*) (L1) Cl]BF_4_ (Ir-1). To the suspension of 3-phenyl-1-pyridin-2-yl-imidazo[1,5-*α*]pyridine L1 (0.1 g, 0.36 mM) in methanol and dichloromethane solution, was added the methanolic solution of [Ir(Cp*)(Cl)_2_]_2_ (0.14 g, 0.184 mM) dropwise and the mixture was stirred at room temperature for 1 h. NH_4_BF_4_ (0.05 g, 0.54 mM) was then added and further stirred overnight. The volatile solvent was removed and the formed residue was washed with dichloromethane and filtered to remove excess salt. Finally, the solvent was reduced and the diethyl ether was added to induce precipitation yielding yellow solid. Yield 82%; ^1^H NMR (400 MHz, DMSO-*d*_6_) δ 8.84–8.82 (d, 1H *J* = 8Hz), 8.54–8.44 (m, 3H), 8.18–8.15 (t, 3H *J* = 6 Hz), 7.75 (s, 3H), 7.56–7.49 (m, 2H), 7.17–7.13 (t, 1H *J* = 8 Hz), 1.27 (s,15H); UV–Vis (λ_abs_, nm) (ε, M^−1^cm^−1^): 401 (1739), 381 (2586), 365 (2043), 284 (2633), 245 (ESI-MS *m*/*z* 634 [M^+^ + BF_4_^−^].

[Ir (Cp*) (L2) Cl]BF_4_ (Ir-2). ^1^H NMR (400 MHz, DMSO-*d*_6_) δ 9.10 (s,1H), 8.86–8.85 (d, 1H *J* = 4 Hz), 8.63–8.51 (m, 6H), 8.20–8.17 (t, 1H *J* = 6 Hz), 8.07–8.03 (t, 1H *J* = 8 Hz), 7.57–7.53 (t, 2H *J* = 8 Hz), 7.21–7.18 (t, 1H *J* = 6 Hz), 1.27 (s, 15H). UV-Vis (λ_abs_, nm) (ε, M^−1^cm^−1^): 400 (943), 378 (1449), 360 (1227), 280 (1601), 246 (1664) ESI-MS *m*/*z* 679 [M^+^ + BF_4_^−^].

[Ir (Cp*) (L4) Cl]BF_4_ (Ir-4). ^1^H NMR (400 MHz, DMSO-*d*_6_) δ 8.82 (s, 1H), 8.51 (s, 3H), 8.17–8.15 (t, 1H *J* = 4 Hz), 7.76–7.75 (d, 2H *J* = 4 Hz), 7.54–7.48 (m, 2H), 7.32–7.29 (m, 1H), 7.14–7.13 (d, 1H *J* = 4 Hz), 3.91 (s, 3H), 3.85 (s, 3H), 1.29 (s, 15H). UV-Vis (λ_abs_, nm) (ε, M^−1^cm^−1^): 406 (1170), 385 (1651), 364 (1217), 285 (2051), 245 (1795) ESI-MS *m*/*z* 694 [M^+^ + BF_4_^−^].

### 4.3. Platelet Aggregation and ATP Release Assay

Healthy men and women (20–35 years old) were engaged for the study. The subjects had no history of severe diseases, such as cardiovascular diseases (CVDs), hypertension, type 1 or type 2 diabetes, thyroid disorders, or hemostatic disorders. The subjects were educated to abstain from taking medication that is known to affect platelet function for a 14-day period before participating in the study. Written informed consent was gained from all subjects. The Institutional Review Board of Taipei Medical University, Taiwan (TMU-JIRB-N201612050; 20 January 2017) was approved this study, and it conformed to the directives of the Declaration of Helsinki.

According to the method described from our previous study, the human platelet suspensions were prepared. Briefly, human blood samples were collected from adult volunteers who abstained from the use of drugs or other substances that could affect with the experiment for at least 14 days before collection; the collected blood samples were mixed with an acid–citrate–dextrose solution. After centrifugation, the platelet-rich plasma was supplemented with 0.5 μM PGE1 and 6.4 IU/mL heparin. Tyrode’s solution containing 3.5 mg/mL BSA was used to prepare the final suspension of washed human platelets. The final Ca^2+^ concentration in Tyrode’s solution was 1 mM. The platelet aggregation study was conducted using a lumiaggregometer (Payton Associates, Scarborough, ON, Canada) [43]. An isovolumetric solvent control (0.1% DMSO) or Ir-1, Ir-2 and Ir-4 complexes were preincubated with platelet suspensions (3.6 × 10^8^ cells/mL) for 3 min before the addition of collagen and U46619. The amount of platelet aggregation was measured as the percent compared with individual control (without Ir compounds) after the reaction proceeded for 6 min and is stated in light transmission units. During the measurement of the ATP release assay, 1 min before adding the collagen (1 µg/mL), 20 μL of luciferin–luciferase was added and the amount of ATP released was equated with that released by the control (without Ir compounds).

### 4.4. Measurement of Relative [Ca^2+^]i Mobilization

The concentration of [Ca^2+^]i was measured using Fura-2AM per the method described previously [42]. Concisely, citrated whole blood was centrifuged at 120× *g* for 10 min, and the platelet rich plasma (PRP) was collected and subjected to 5 μM Fura-2AM for 1 h. As described in the above section, human platelets were prepared. The Fura-2AM-added platelets were pretreated with 10 µM Ir-1, Ir-2 and Ir-4 in the presence of 1 mM CaCl_2_ and then stimulated with 1 µg/mL of collagen. Using a spectrofluorometer (Hitachi FL Spectrophotometer F-4500, Tokyo, Japan), the Fura-2 fluorescence was measured at excitation wavelengths of 340 and 380 nm and an emission wavelength of 510 nm.

### 4.5. Flow Cytometric Analysis

The platelet suspensions (3.6 × 10^8^ cells/mL) were preincubated with 0.1% DMSO (solvent control) or 10 µM of each Ir-1, Ir-2 and Ir-4 and FITC-P-selectin (2 µg/mL) for 3 min, and 1 µg/mL collagen was added to trigger platelet activation. In other experiment, the FITC labeling of the anti-GPVI mAb (FITC-JAQ1 mAb) was done, in which the platelets (3.6 × 10^8^ cells/mL) were preincubated with 10 ng/mL of convulxin or 20 μM Ir-1 for 3 min, followed by the addition of 1 μg/mL of FITC-JAQ1 mAb. For isotype controls, FITC-IgG antibody (Biolegend, Cat. No. 400108) was used. The suspensions were then assayed for fluorescein-labeled platelets by using a flow cytometer (FACScan System, Becton Dickinson, San Jose, CA, USA). Data were collected from 50,000 platelets per experimental group, and the platelets were identified with their characteristic forward and orthogonal light-scattering profiles. All experiments were repeated at least three times to confirm reproducibility.

### 4.6. Detection of Lactate Dehydrogenase (LDH)

Washed platelets (3.6 × 10^8^ cells/mL) were preincubated with 0.1% DMSO or 100 μM each Ir-1, Ir-2 and Ir-4 for 20 min at 37 °C. A 10 µL of the supernatant was deposited on a Fuji Dri-Chem slide LDH-PIII (Fuji, Tokyo, Japan), and the optical density was read at 540 nm wavelength by using a UV-Vis spectrophotometer (UV-160; Shimadzu, Japan). “Max” is considered as maximal value of LDH recorded in the sonicated platelets.

### 4.7. Immunoblotting

Washed platelets (1.2 × 10^9^ cells/mL) were pre-incubated with 10 μM Ir-1, Ir-2 and Ir-4 or 0.1 % DMSO for 3 min and then collagen was added to induce platelet activation. After the reaction stopped, the platelets were directly re-suspended in 200 μL of lysis buffer. Samples (60 μg protein) were resolved on a 12% sodium dodecylsulfate polyacrylamide gel electrophoresis (SDS-PAGE), and transferred to the PVDF membranes by using a Bio-Rad semidry transfer unit (Hercules, CA, USA). Membranes were blocked with TBST (10 mM Tris-base, 100 mM NaCl, and 0.01% Tween 20) containing 5% BSA for 1 h and probed with various primary antibodies. HRP-conjugated anti-mouse IgG or anti-rabbit IgG (diluted 1:3000 in TBST) antibodies were used and the bands were visualized using an enhanced chemiluminescence system.

### 4.8. Measurement of Closure Time Using PFA-100™ Platelet Function Analyzer

The platelet functions were analyzed using a Dade Behring PFA-100 system (Dade Behring, Marburg, Germany) according to a method described [44]. First, 0.8 mL of human whole blood was treated with Ir-1, Ir-2, Ir-4 (10 µM) or the solvent control (0.1% DMSO) for 2 min, and then poured onto the cartridges containing collagen-ADP (C-ADP) or collagen epinephrine (C-EPI)-coated membranes, and subjected to a high shear rate of 5000–6000/s. A platelet plug forms, then slowly occludes the aperture; subsequently, the blood flow gradually decreases and finally stops. The time required to obtain full occlusion of the aperture by the platelet clot is defined as the “closure time” and was recorded in the collagen membrane [44].

### 4.9. Confocal Microscopic Analysis of Platelet Adhesion and Spreading

Eight-chamber glass coverslides were coated with BSA (100 μg/mL), fibrinogen or collagen (100 μg/mL) at 4°C overnight. The coverslides were blocked with 1% BSA for 1 h after being washed twice with PBS. Washed platelets (3.6 × 10^8^ cells/mL) preincubated with Ir-1 (10 μM) or the solvent control (0.1% DMSO) were allowed to spread on the protein-coated surfaces at 37 °C for 45 min. After removing the unbound platelets and two washes with PBS, the attached cells were fixed with 4% paraformaldehyde, permeabilized (0.1% Triton) and stained with FITC-labeled phalloidin (10 μM) for 1 h. The confocal study was done with a Leica TCS SP5 microscope furnished with a 100×, 1.40 NA oil immersion objective (Leica, Wetzlar, Germany).

### 4.10. Animals

ICR mice (20–25 g, male, 5–6 weeks old) were obtained from BioLasco (Taipei, Taiwan). All procedures were approved by the Affidavit of Approval of Animal Use Protocol-Taipei Medical University (LAC-2016-0395) and were in accordance with the Guide for the Care and Use of Laboratory Animals (8th edition, 2011). Since among the three tested Ir-III complexes, Ir-1 (10 µM) effectively inhibits platelet activation in vitro, this concentration was chosen and calculated accordingly into mouse dose 2 mg/kg [45].

### 4.11. Fluorescein Sodium-Induced Platelet Thrombi in Mesenteric Microvessels of Mice

This study conformed to the Guide for the Care and Use of Laboratory Animals (Approval No. LAC-2016-0395; 1 August 2017). As described previously [46], the jugular vein was exposed and cannulated using PE-10 tubing to administer the dye and compounds (by an i.v. bolus). Vessels (30–40 μm) were selected for irradiation (below 520 nm) to form a microthrombus. One minute after fluorescein sodium (15 μg/kg) given, Ir-1, Ir-2 and Ir-4 (2 mg/kg) were administered. The time lapse for inducing thrombus formation leading to cessation of blood flow was measured.

### 4.12. Measurement of Bleeding Time in Mouse Tail Vein

Experiments were performed through transection of the tails in male ICR mice. Briefly, after an intraperitoneal administration of 2.0 mg/kg Ir-1, Ir-2 and Ir-4 for 30 min, the mice tails were cut 3 mm from the tip. The tails were immersed in normal saline at 37 °C, and the time from incision to full cessation of bleeding was recorded.

### 4.13. Acute Pulmonary Thrombosis Induced by ADP in Mice

A dose of 2 mg/kg of Ir-1, Ir-2 and Ir-4 and vehicle solution (0.1 % DMSO, all in 20 μL) were injected into the tail vein of 20–25 g ICR mice for 30 min, followed by 700 mg/kg ADP injection into the contralateral vein [47]. The mortality rate was determined in mice of each group within 5 min after injection.

### 4.14. Statistical Analysis

The experimental data are produced as the mean ± standard error of the means (S.E.M.) and are presented by the number of observations (*n*). The unpaired Student *t* test was used to analyze the significance of variations between the control and experimental mice. The differences between multiple groups in other experiments were assessed through one-way analysis of variance (ANOVA). When ANOVA showed significant differences among the group means, the groups were compared using the Student Newman–Keuls method. In the analysis, *p*-values < 0.05 were considered statistically significant. Statistical evaluations were performed using SAS (version 9.2; SAS Inc., Cary, NC, USA).

## 5. Conclusions

In this work, we showed that, among the three newly synthesized organometallic iridium-III complexes, Ir-1, Ir-2 and Ir-4, Ir-1 has a promising in vitro antiplatelet and in vivo antithrombotic profiles. Ir-1 actively inhibits collagen-stimulated platelet activation by inhibiting the Akt/PKC pathways, and subsequently by suppressing activation of MAPKs. These alterations reduce the levels of surface P-selectin expression and [Ca^2+^]i, and ultimately inhibit platelet aggregation. Our SAR study suggested that the inhibitory activity of Ir-1 is modulated by its increasing hydrolytic nature. Altogether, these results suggest that Ir-1 may be a leading novel Ir-III complex to design new antiplatelet drugs for the treatment of thromboembolic diseases.
